# The quality of creep feed affects consumption and growth performance of pre- and post-weaning pigs

**DOI:** 10.1093/tas/txaf126

**Published:** 2025-09-18

**Authors:** Rommel C Sulabo, Jamil E G Faccin, Mike D Tokach, Jon R Bergstrom, Joel M DeRouchey, Robert D Goodband, Steve S Dritz

**Affiliations:** Department of Animal Sciences and Industry, Kansas State University, Manhattan, KS 66506-0201, United States; Department of Animal Sciences and Industry, Kansas State University, Manhattan, KS 66506-0201, United States; Department of Animal Sciences and Industry, Kansas State University, Manhattan, KS 66506-0201, United States; Department of Animal Sciences and Industry, Kansas State University, Manhattan, KS 66506-0201, United States; Department of Animal Sciences and Industry, Kansas State University, Manhattan, KS 66506-0201, United States; Department of Animal Sciences and Industry, Kansas State University, Manhattan, KS 66506-0201, United States; Department of Diagnostic Medicine/Pathobiology, College of Veterinary Medicine, Kansas State University, Manhattan, KS 66506-0201, United States

**Keywords:** creep feeding, feed quality, growth, nursery pigs

## Abstract

Two experiments were conducted to evaluate the effects of creep feeding on pre- and post-weaning pig performance. In Exp. 1, 96 sows (C29, PIC) and their litters were used to determine the effects of creep feed quality on pre-weaning growth and the proportion of piglets consuming creep feed. Litters were offered one of three treatments: (i) no creep feed, (ii) a simple creep diet, or (iii) a complex creep diet. The simple creep feed was the sow lactation diet which was sorghum-soybean meal based and formulated to 2554 kcal/kg NE and 0.92% standardized ileal digestible (SID) Lys. The complex creep feed was formulated to 2769 kcal/kg of NE, 1.44% SID Lys, 23% lactose, and composed of oat groats, dried whey, and specialty protein sources. Creep feed was offered from d 18 to 21 (weaning) of lactation. Chromic oxide was added to both diets at 1.0% as an inert, colored fecal marker to identify pigs that consumed (eaters) and did not consume creep feed (non-eaters). Pigs fed the complex creep diet had greater (*P *= 0.033) pre-weaning ADG and tended to have greater (*P *= 0.063) total gain than pigs fed the simple creep diet with pigs not offered a creep diet intermediate. Litters fed the complex creep diet consumed twice (*P *< 0.001) as much total and daily creep feed intake of litters fed the simple creep diet. Also, the complex creep diet improved (*P *< 0.001) the proportion of eaters from 28% to 68%. In Exp. 2, 675 pigs from Exp. 1 (initially 6.4 ± 0.13 kg; 21.2 ± 0.2 d) were used to evaluate whether placing eaters with non-eaters of creep feed would facilitate and increase intake and gain of non-eaters. Treatments were non-eaters (not provided any creep feed or non-eaters from creep fed litters), eaters (pigs that consumed the complex creep feed), and a mixed group (52% non-eaters and 48% eaters). Each treatment had 9 replicate pens with 25 pigs per pen. In the first 3 d, eaters had greater (*P *< 0.05) average daily gain (ADG) and average daily feed intake (ADFI) than non-eaters with the mixed group intermediate. Overall (d 0 to 28), ADG of eaters was greater (*P *= 0.049) than non-eaters. In conclusion, the complex creep improved pre-weaning ADG, ADFI, and the proportion of eaters. Eaters had increased ADG and ADFI in the first 3 d post-weaning and greater overall nursery ADG. Additionally, mixing eaters with non-eaters within pens did not stimulate feed intake and growth of non-eaters, which indicates that social facilitation did not occur.

## Teaser

Providing a complex creep feed to piglets increases pre-weaning growth and feed intake compared with those not offered creep feed or offered a simple creep feed. Pigs that ate creep feed before weaning grew faster after weaning; however, mixing piglets that consumed creep feed with those that did not in the nursery did not improve the growth of non-eaters, suggesting that social facilitation did not occur.

## Introduction

Maximizing post-weaning pig performance is important for improving lifetime growth performance and survivability (Faccin et al 2020). However, weaning is often characterized by a period of low feed intake caused by physical, physiological, and behavioral challenges that may result in a growth check and affect post-weaning growth rates (Li and Patience 2017; Moeser et al 2017; Wensley et al 2021). Creep feeding studies that evaluated individual pigs rather than whole litters demonstrated that pigs that consumed creep feed have greater post-weaning feed intake and growth than non-eaters (Sulabo et al 2010b, 2010c; Muns and Magowan 2018; Wensley et al 2023). Therefore, identifying factors that can increase the proportion of eaters is necessary for improving the success of this practice.

It is hypothesized that the quality of creep feed is an essential factor. Fraser et al (1994) and Pajor et al (2002) observed improvements in pre- and post-weaning feed intake in litters fed a high-quality creep feed. However, there is limited research evaluating the effects of creep feed complexity on individual pig consumption characteristics. Additionally, specialized complex creep diets are usually produced at different feed mills than the manufacturer of the diets for gestating and lactating sows. Thus, the use of complex nursery diets can be logistically difficult and increase biosecurity risk. To easily facilitate creep feeding in commercial production, offering the lactation diet would be simple, easy, and an efficient means of providing creep feed to litters. It is also speculated that weaned pigs with pre-weaning experience to solid food may facilitate non experienced pigs to discover food sources and initiate feeding when housed together in large nursery groups (Weary et al 2008). However, evidence of this social learning behavior is limited.

Therefore, our hypothesis was that creep feed quality affects the proportion of pigs consuming creep feed and their respective pre-weaning performance, and that social facilitation occurs between eaters and non-eaters, improving post-weaning feed intake and performance in the nursery.

## Materials and methods

The protocol used in this experiment was approved by the Kansas State University Institutional Animal Care and Use Committee (2767). Two experiments were conducted to evaluate the effects of creep feeding on pre- and post-weaning pig performance in a commercial environment.

### Experiment 1

A total of 96 sows (C29, PIC, Hendersonville, TN) and their litters were used in this study conducted at a commercial sow farm in northeast Kansas. Sows used in this experiment were from 3 batches farrowed during the same month. On d 18 of lactation, sows were allotted to 3 experimental treatments using a randomized complete block design with parity, date of farrowing, and litter size used as blocking factors. For treatment 1, litters were not provided any creep feed (control). For treatments 2 and 3, litters were provided either a simple or a complex creep feed, respectively. There were 26 replicates for treatments 1 and 2 and 44 replicates for treatment 3 which was intended to generate a greater number of eaters that would be used for Exp. 2.

Two creep diets were formulated that differed in complexity, quality of feed ingredients, nutrient specifications, and physical form of the feed (Table 1). The simple creep feed was the farm’s lactation diet containing sorghum and soybean meal with 3% choice white grease and formulated to contain 2554 kcal/kg NE, 20.8% CP, 0.92% standardized ileal digestible (SID) Lys, 1.00% Ca, and 0.55% STTD P. The simple creep feed was fed in meal form. In contrast, the complex creep feed was a pelleted diet composed of pulverized oat groats and milk-based products that included spray-dried whey and lactose. Extruded soy protein concentrate, spray-dried animal plasma, and select menhaden fish meal were included to provide high concentrations of digestible CP and AA. The inclusion of corn and soybean meal were limited to 6.25% and 2.32%, respectively. This creep feed was also high in added fat with 5% choice white grease. Feed additives such as antibiotics, zinc oxide, and acidifier (calcium propionate) were also included in the diet. The diet was formulated to contain 2769 kcal/kg NE, 1.44% SID Lys, 0.77% Ca, 0.56% STTD P, and 23% lactose and it was fed in 2-mm diameter by 6-mm length pellets. Chromic oxide was added to both diets at 1.0% to serve as an inert, colored fecal marker to identify individual pigs that consumed or did not consume creep feed.

**Table 1.** Experimental diets (as-fed basis), Exp. 1.

Item	Simple creep feed^a^	Complex creep feed^b^
**Ingredient, %**		
**Corn**	–	6.25
**Milo**	60.40	–
**Soybean meal, 46.5% CP**	31.65	2.32
** Spray-dried whey**	–	25.00
** Fine ground oat groats**	–	30.00
** Extruded soy protein concentrate**	–	10.00
** Spray-dried porcine plasma**	–	6.00
** Select menhaden fish meal**	–	6.00
**Lactose**	–	5.00
** Choice white grease**	3.00	5.00
** Monocalcium P, 21% P**	1.35	0.35
** Chromium oxide**	1.00	1.00
** Antibiotic^c^**	–	1.00
** Limestone**	1.35	0.40
** Zinc oxide**	–	0.38
**Salt**	0.50	0.30
** L-Lysine HCl**	–	0.15
**DL-methionine**	–	0.15
** Trace mineral premix^d^**	0.15	0.15
** Vitamin premix^e^**	0.25	0.25
** Sow add pack^f^**	0.25	–
** Acidifier^g^**	–	0.20
** Phytase^h^**	0.10	–
** Vitamin E, 20,000 IU**	–	0.05
**Calculated composition**		
** CP, %**	20.8	23.1
** SID Lys, %^i^**	0.92	1.44
** NE, kcal/kg**	2554	2769
** SID Lys:NE, g/Mcal**	3.61	5.19
** Calcium, %**	1.00	0.77
** STTD P, %**	0.55	0.55
** Lactose, %**	–	23.0

a Diet fed in meal form.

b Diet fed in pellet form (2-mm by 6-mm pellets).

c Contained 35 mg of Denagard (Elanco, Innovation Way Greenfield, IN) and 400 mg of Chlortetracycline per kg of complete diet.

d Provided per kg of complete diet: 16.5 mg Cu; 165.4 mg Fe; 39.7 mg Mn; 0.30 mg Se; 165.4 mg Zn; and 0.30 mg I.

e Provided per kg of complete diet: 11,023 IU vitamin A; 1378 IU vitamin D; 44 IU vitamin E; 4 mg vitamin K (as menadione dimethylpyrimidinol bisulfate); 50 mg niacin; 28 mg pantothenic acid (as d-calcium pantothenate); 8 mg riboflavin; and 0.04 mg vitamin B12.

f Sow add pack provided the following nutrients per kg of complete diet: 22 IU vitamin E; 0.22 mg biotin; 1.65 mg folic acid; 5 mg pyridoxine (as pyridoxine HCl); 551 mg choline (as choline Cl); 50 mg L-carnitine; and 0.20 mg chromium (as chromium picolinate).

g Calcium propionate.

h Provided 750 FYT of Ronozyme P phytase (DSM Nutritional Products, Parsippany, NJ).

i Standardized ileal digestible.

Creep feeds were offered from d 18 until weaning on d 21. A rotary creep feeder with a hopper (Rotecna Mini Hopper Pan, Rotecna SA, Agramunt, Spain) was used and placed on the opposite side of the farrowing crate from the heat lamp. The feeder has a 6-L capacity hopper, which is adjustable to 5 different feeder gap settings. The feeder was checked daily to ensure ad libitum access and minimize feed wastage. A single lactation diet (2518 kcal/kg NE, 0.97% SID Lys) was used. Sows were allowed free access to feed throughout lactation. Water was available ad libitum for sows and their litters through nipple and bowl drinkers, respectively.

Piglets were individually weighed at d 0 (birth), 18 (start of creep feeding), and 21 (weaning). The creep feed was placed in the hopper of the creep feeder at the start of the study (d 18), and the initial weight of the creep feeder was weighed and recorded. Feeders were weighed daily to calculate daily and total creep feed disappearance for each litter. Individual consumption characteristics of pigs in creep fed litters were determined using procedures adapted from Barnett et al (1989) and Bruininx et al (2002). On the morning of d 20, an individual fecal swab was obtained from each piglet from creep fed litters, and the piglet was categorized as an eater if the green color was visible in the fecal sample. Piglets that tested negative on the first fecal sampling day were resampled 3 to 12 h before weaning on d 21. When no green color was detected in the final sample, the piglet was categorized as a non-eater. The general health of the sows and piglets was checked daily by farm employees. Temperature in the farrowing facility was maintained at a minimum of 20 °C, and supplementary heat was provided to the piglets with heat lamps.

The relationship between creep consumption category and teat suckled was also determined. Teat order was defined as the specific teat (pair) nursed by each piglet with respect to the anatomical location of the nursed mammary gland (Kim et al 2000). Individual pigs categorized as eaters were marked on their back, and non-eaters were unmarked. On d 20 (within 24 h before weaning), a suckling bout from 20 litters were photographed using a digital camera (Sony Cybershot DSC W55). Litters with less than 50% eaters were selected to obtain a distribution of eaters and non-eaters. The photograph of each suckling bout was then used to determine teat location and rank of each individual piglet in the litter. A distribution of teat order in three classes was made based on the preferred teat pair suckled by the piglets: anterior (teat pairs 1 and 2), middle (teat pairs 3, 4, and 5), and posterior (teat pairs ≥ 6).

### Experiment 2

At weaning, pigs were transported to an off-site nursery approximately 3.6 km from the sow farm. From a total of 1024 pigs weaned in Exp. 1, 675 pigs (initially 6.4 ± 0.13 kg and 21.2 ± 0.2 d, C29 × 327, PIC) were allotted to three treatments using a randomized complete block design with initial weight as the blocking factor. The experimental treatments were as follows: Treatment 1 was pens of pigs provided no creep feed and pigs that did not consume creep feed when offered (non-eaters); Treatment 2 was pens of pigs that consumed creep feed (eaters), and Treatment 3 were pens with 13 non-eaters and 12 eaters (mixed pens). Each pen had 25 pigs and there were 9 replicate pens per treatment. Pens were equipped with a 10-hole self-feeder (Farmweld, Inc., Teutopolis, IL) and 1 cup drinker to provide ad libitum access to feed and water.

After weaning, pigs were fed 454 g per pig of a commercial pelleted diet formulated to 1.56% SID Lys containing 25% dried whey, 6.7% spray-dried animal plasma and 6% select menhaden fish meal. After the budget was consumed, pigs were then fed 900 g of a second diet formulated to 1.51% SID Lys containing 25% dried whey, 2.5% spray-dried animal plasma, 2.5% spray-dried blood cells, and 2.5% select menhaden fish meal. Pigs were then fed a corn-28% soybean meal diet formulated to 1.35% SID Lys containing 3% select menhaden fish meal, and 1.25% spray-dried blood cells until the end of the study (d 28 post-weaning). The total amount of feed offered in the first 3 d post-weaning was recorded and, to determine total and daily feed intake in the initial 3 d, feed was vacuumed out of the feeders and weighed. Pigs were weighed at d 0 (weaning), 3, 7, and 28 post-weaning to calculate ADG.

### Statistical analyses

In Exp. 1, data were analyzed using the MIXED procedure of SAS (SAS Institute Inc., Cary, NC) with litter as the experimental unit and the model included treatment as the fixed effect and block as the random effect. To determine the effects of creep feed consumption within creep-fed litters, the model included the main effects of creep feed quality (simple or complex) and consumption category (Y or N) and their interaction as the fixed effects and block as the random effect, with pig as the experimental unit. To determine the effects of consumption within weight categories, the model included the main effects of consumption and weight category and their interaction as the fixed effects and block as the random effect; pig was the experimental unit. Pigs were divided into three body weight (BW) categories: top ≥ least squares mean + 1 SD, middle = least squares mean ± 1 SD, and bottom ≤ least squares mean - 1 SD. The effects of creep feed quality, weight category, and teat location on the proportion of eaters were analyzed using the χ^2^-square test.

In Exp. 2, growth performance data were analyzed using the MIXED procedure of SAS with pen as the experimental unit. The model included consumption category (Y or N) and block as the fixed and random effects, respectively. To test the evidence of social facilitation, the effect of consumption was analyzed within the mixed pens using the MIXED procedure of SAS with pig as the experimental unit. The model included consumption (Y or N) as the fixed effect and pen as the random effect. Least squares means were calculated for each independent variable. Statistical significance and tendencies were set at *P *≤ 0.05 and *P *< 0.10 for all analysis.

## Results and discussion

### Experiment 1

Sows had an average parity of 4.3 ± 0.4 and lactation length of 21.2 ± 0.2 d. The average litter size at d 18 and 21 (weaning) was 10.7 ± 0.3 and 10.5 ± 0.3 piglets, respectively (Table 2). Mortality rate during the creep feeding period (d 18 to 21) was 1.9% and unaffected by treatment. There were no differences in pig BW at weaning; however, pigs fed the complex creep feed had greater total gain (*P *< 0.063) and ADG (*P *< 0.033) during the creep feeding period than pigs fed the simple creep feed, with no-creep fed pigs intermediate. The improvement in ADG from d 18 to 21 wasn’t great enough to influence overall litter gain, ADG, or BW at weaning.

**Table 2.** Effects of creep feed quality on pig and litter performance.^a^

Item	No creep	Creep feed quality		SEM	*P*-value
		Simple	Complex		
**No. of litters**	26	26	44	…	…
**No. of pigs/litter**					
** d 18 (creep start)**	10.8	11.0	10.3	0.30	0.301
** d 21 (weaning)**	10.5	10.8	10.2	0.30	0.386
**Weaning age, d**	21.3	21.2	21.2	0.20	0.866
**Pig BW, kg**					
** d 0 (post-fostering)**	1.56	1.53	1.58	0.06	0.704
** d 18 (creep start)**	5.68	5.64	5.65	0.20	0.958
** d 21 (weaning)**	6.44	6.37	6.45	0.21	0.745
** Total gain (d 18 to 21), g**	758^bc^	721^c^	800^b^	29	0.063
** ADG (d 18 to 21), g**	253^bc^	240^c^	267^b^	10	0.033
**Litter BW, kg**					
** d 0 (post-fostering)**	16.53	16.80	16.35	0.87	0.908
** d 18 (creep start)**	59.83	60.78	57.87	3.02	0.605
** d 21 (weaning)**	67.66	68.51	65.87	3.27	0.702
** Total gain (d 18 to 21), kg**	7.82	7.72	7.99	0.33	0.791
** ADG (d 18 to 21), kg**	2.61	2.58	2.66	0.12	0.793

a Three groups of sows (total = 96, PIC; avg. parity = 4.3 ± 0.4) were blocked according to parity, farrowing day, and litter size and allotted to 3 treatments: no creep = litter was not provided any creep feed, a simple, and a complex creep feed. Data were analyzed with litter as the experimental unit. Creep feed with 1.0% chromic oxide was offered ad libitum from d 18 to weaning (21 d) using a rotary feeder with a hopper.

b,c Row values (LSM ± SEM) with different superscripts differ (*P *< 0.05).

Previously, Fraser et al (1994) provided creep feed to piglets from d 14 of lactation until weaning at d 28 and compared a ‘low-quality’ diet based on corn, barley, and soybean meal with a ‘high-quality’ diet. Their results showed a tendency for greater ADG during the week before weaning and heavier weaning weights in pigs fed the high-quality creep feed. The results of the current study support these findings, in which an improved quality diet increased pre-weaning BW gains even with different creep feeding duration and weaning age. The complex creep feed was formulated to match the digestive capacity of young pigs, where digestibility, palatability, and antigenic properties of the feed were considered. These same requirements were disregarded in the design of the simple creep feed (sow lactation diet). However, the lack of differences in overall pig and litter pre-weaning gains between the creep-fed and no-creep fed pigs may suggest that the amount of feed intake is not enough to generate an improvement in overall piglet BW gain. This result was also observed by other studies with different creep feed duration (Sulabo et al 2010c; Huting et al 2017; Middelkoop et al 2020). On the other hand, ­Wensley et al (2023) observed improvements in weaning weight with creep feeding, but the authors highlighted a tendency of an interaction between creep feed and genetic line, indicating that different genetics might respond differently to creep feed.

**Fig. 1.**
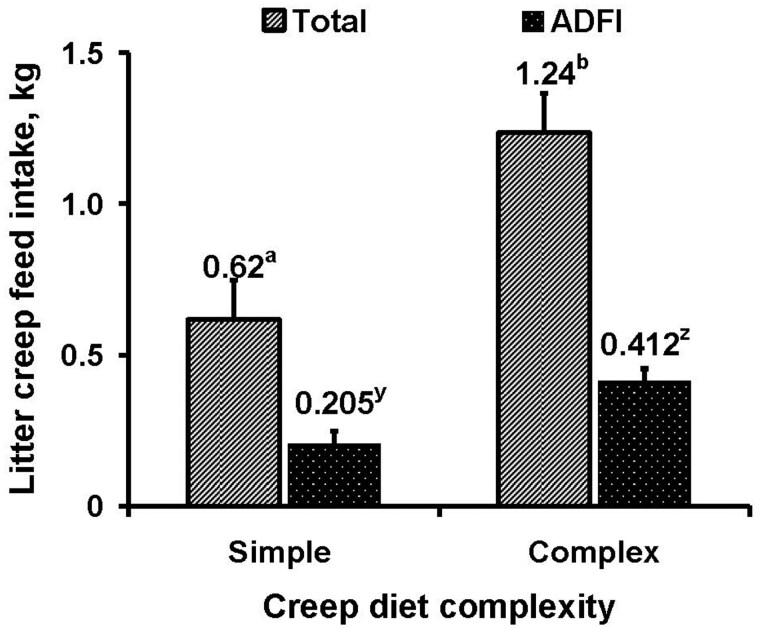
Total and daily creep feed intake of litters fed either simple or complex creep diets. ^a,b^*P* < 0.001; ^y,z^*P* < 0.001.

Litters fed the complex creep feed consumed twice the total (*P *< 0.001) and daily (*P *< 0.001) amount of creep feed compared to litters provided the simple creep feed (Fig. 1). Consequently, piglets fed the complex creep had increased total gain and ADG (*P *< 0.05) than those provided the simple diet (Table 3). Similarly, Fraser et al (1994) observed that piglets consumed about twice the amount of a high-quality creep feed than those fed a low-quality feed. Pajor et al (2002) also reported that pigs fed a complex creep feed high in CP and fat consumed 52% more than pigs fed a standard creep feed. However, more recently, Sands et al (2022) offered simple and complex creep feed with similar SID Lys to the present study for 14 d prior to weaning at d 28 and found similar creep consumed regardless of the creep quality and piglets fed either creep diet had increased ADG from d 21 to 28 as compared with those not provided creep feed. Thus, for pigs weaned at older ages, creep feed quality might not be as important as for younger pigs.

**Table 3.** Interactive effects of creep feed quality and consumption category on preweaning performance of creep-fed pigs.^a^

Item	Simple		Complex		SEM	*P*-value		
	Non-eater	Eater	Non-eater	Eater		Quality × Consumption	Quality	Consumption
**N**	203	79	145	304	…	…	…	…
**Pig BW, kg**								
** d 0 (post-fostering)**	1.53	1.52	1.57	1.56	0.06	0.878	0.621	0.632
** d 18 (creep start)**	5.74	5.27	5.93	5.52	0.20	0.785	0.403	<0.001
** d 21 (weaning)**	6.47	5.96	6.75	6.28	0.21	0.824	0.295	<0.001
**Total gain, g**	734	685	814	774	3	0.842	0.027	0.084
**ADG, g**	244	228	271	259	11	0.931	0.029	0.092

a Three groups of sows (total = 96, C29 PIC; avg. parity = 4.3 ± 0.4) were blocked according to farrowing day and allotted to three treatments: no creep = litter was not provided any creep feed, simple = litter was provided a simple creep diet, complex = litter was provided a complex creep diet. In the simple and complex treatments, individual pigs were sampled at d 19 and 20 using fecal swabs to determine consumption category. A pig was categorized as an eater when it showed green-colored feces in at least 1 of the 2 samplings and as a non-eater when all samples were negative for green-colored feces. Data were analyzed with pig as the experimental unit. Creep feed with 1.0% chromic oxide was offered ad libitum from d 18 to weaning (21 d) using a rotary feeder with a hopper.

**Fig. 2.**
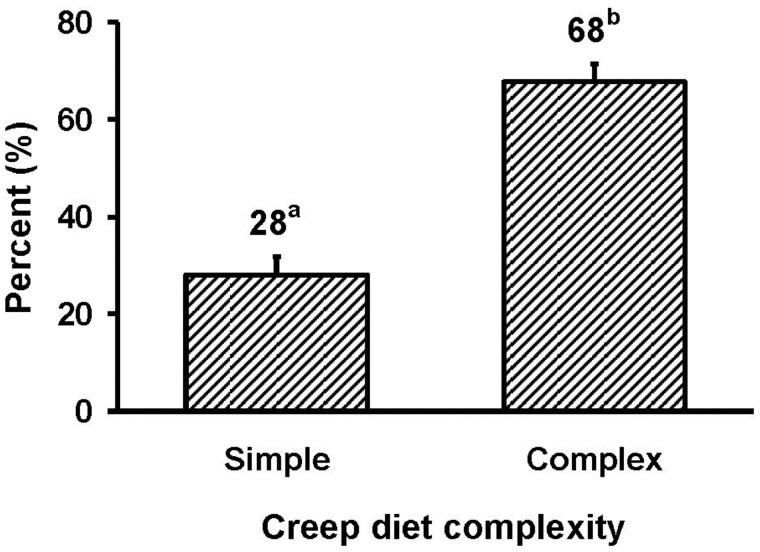
Effect of creep diet complexity on the proportion of eaters in whole litters. ^a,b^*P* < 0.001.

Creep feed quality also affected the proportion of pigs in the litter consuming creep feed with a greater (*P < *0.001) proportion of pigs consuming creep feed when provided complex creep diet as compared to a simple creep diet (Fig. 2). The proportion of eaters achieved in this study for the complex creep feed was consistent with previous studies in which the same complex creep diet, feeder design, and creep feeding duration were used (Sulabo et al 2010a, 2010c, 2010d). The diet composition and diet form may both be partially responsible for the increase in number of eaters in this study with the complex creep diet. In exploring diet form, Christensen and Huber (2021) found that a liquid milk replacer generated more eaters than a pelleted milk replacer or a commercial creep diet. Craig et al (2021) suggested that pellet size can influence creep feed intake with a larger, 9 × 12 mm pellet increasing intake compared to a smaller, 4 × 4 mm pellet. This indicates that the creep feed quality and form may be critical factors in stimulating individual pigs in the litter to consume creep feed.

Pigs that became eaters in creep-fed litters were lighter (*P *< 0.001) at d 18 and weaning regardless of the creep feed quality (Table 3). Eaters also tended to have lower (*P *= 0.084) total gain than non-eaters. The ADG of eaters was approximately 6.0% lower than those of non-eaters; however, differences were not significant. There were significant differences (*P *< 0.001) in pig weights at d 18 and at weaning, total gain, and ADG between the bottom, middle, and top weight categories for pigs fed either the simple or complex creep feed (Table 4). A greater percentage (*P *< 0.001) of eaters were observed among pigs in the bottom weight category for both creep-fed treatments (47% and 83% in the simple and complex creep feed, respectively). Thus, these results suggest that creep feeding is beneficial to smaller piglets within litters as an alternative source of nutrients during lactation.

**Table 4.** Effects of creep feed quality on suckling pig performance according to weight category.^a^^,^^b^

Item	Simple			SEM	Complex			SEM	*P*-value^c^
	Bottom	Middle	Top		Bottom	Middle	Top		
**N**	45	198	39	…	81	301	67	…	…
**% of total**	16.0	70.2	13.8	…	18.0	67.0	14.9	…	…
**% eaters**	46.7	24.7	23.1	…	82.7	65.1	62.7	…	…
**Pig BW, kg**									
** d 18 (start creep)**	4.10	5.64	7.18	0.10	3.67	5.87	7.91	0.09	<0.001
** d 21 (weaning)**	4.62	6.37	8.06	0.12	4.32	6.68	8.80	0.09	<0.001
**Total gain, g**	517	729	886	38.1	650	813	893	33.0	<0.001
**ADG, g**	172	243	295	14.6	217	271	297	12.0	<0.001

a Three groups of sows (total = 96, C29, PIC; avg. parity = 4.3 ± 0.4) were blocked according to farrowing day and allotted to three treatments: no creep = litter was not provided any creep feed, simple = litter was provided a simple creep diet, complex = litter was provided a complex creep diet. Creep feed with 1.0% chromium oxide was offered ad libitum from d 18 to weaning (21 d) using a rotary feeder with a hopper. Data were analyzed with pig as the experimental unit.

b Weight categories for each population: top ≥ least squares mean + 1 SD, middle = least squares mean ± 1 SD, bottom ≤ least squares mean - 1 SD.

c No creep quality × weight category interaction detected (*P *≥ 0.05).

One of the goals of the creep feeding strategy is to help pigs to achieve nutrient intake other than through sows’ milk. The results found in the present study illustrated which pigs have a greater chance of becoming an eater. Eaters were lighter regardless of the creep diet quality. Interestingly, a tendency (*P *< 0.05) for a greater proportion of eaters nursing in the middle and posterior teats (57% and 52%, respectively) than in the anterior teats (38%) was observed (Table 5). Brooks and Tsourgiannis (2003) suggested that teat order may be related to creep feed consumption, wherein pigs nursing in the posterior (less productive) teats may consume creep feed more readily than their counterparts nursing in anterior (more productive) teats to partially compensate for their lower nutrient intake. However, the literature is contradictory, as Pluske et al (2007) observed no differences in the eaters between pigs nursing anterior or posterior teats, which contrasts the results of the current study. Typically, piglets that nurse from the posterior teats are smaller and less competitive than those that nurse from anterior teats (Pluske and Williams 1996; Kim et al 2000). The lower ability of smaller pigs to compete at the udder and extract milk may predispose these pigs to consume more creep feed when it is offered. The higher rate of eaters in the middle and posterior teats in the current study supports this assumption.

**Table 5.** Proportion of eaters and non-eaters according to teat location.^a^

Teat location	Consumption	
	Non-eater	Eater
**Number of pigs/percentage**		
**Anterior**	35/62.5	21/37.5^b^
**Middle**	30/43.5	39/56.5^c^
**Posterior**	13/48.1	14/51.9^c^

a Eaters in a litter were marked, and non-eaters were unmarked. Suckling bouts (n = 20 litters) were photographed within 24 h before weaning to determine each individual pig’s preferred teat (or pair) at d 21 of lactation. Anterior = teat pairs 1 and 2; middle = teat pairs 3, 4, and 5; posterior = teat pairs ≥ 6. Chi-square test: *P *< 0.10.

b ^,c^Column values (LSM ± SEM) with different superscripts differ (*P *< 0.05).

### Experiment 2

The initial weight of pigs in eater pens (at d 21) was numerically (*P *> 0.05) lower than pigs in non-eater or mixed pens because it was a characteristic of the population of eaters weaned from Exp. 1 (Table 6). Although nursery BW was not affected by consumption category, pigs in eater pens were numerically 3% heavier (15.46 kg vs. 15.02 kg; *P *= 0.146) than pigs in the non-eater group at 49 d of age (last day of the experiment). In the initial 3 d post-weaning (d 21 to 24), eaters had 43% greater (*P *= 0.018) ADG than non-eaters with pigs in mixed pens intermediate. This was mainly due to differences in initial feed intake between groups. The eater group had greater (*P *< 0.002) ADFI than non-eater and mixed groups and the mixed group also had higher (*P *= 0.020) ADFI than the non-eater group. There were no differences in G: F between the 3 groups during the initial 3-d period. From d 3 to 7 (d 25 to 28) and the first 7 d post-weaning (d 21 to 28), there were no differences in ADG between the 3 groups. From d 29 to 49, the eater group tended (*P *= 0.075) to have greater ADG than the non-eater group, with the mixed group intermediate. Overall, ADG of the eater group was 6.2% greater (*P *= 0.049) than that of the non-eater group, with the mixed group intermediate.

**Table 6.** Growth performance of nursery pigs based on individual creep consumption.^a^

Item	Consumption category			SEM	*P*-value		
	Non-eater (N)	Eater (E)	Mixed (M)		N vs. E	N vs. M	E vs. M
**Pig BW, kg**							
** d 21 (weaning)**	6.40	6.33	6.44	0.13	0.411	0.970	0.428
** d 24**	6.70	6.75	6.82	0.12	0.522	0.138	0.348
** d 28**	7.43	7.57	7.47	0.18	0.724	0.246	0.395
** d 49**	15.02	15.46	15.39	0.42	0.146	0.214	0.803
**ADG, g**							
** d 21 to 24**	97	139	125	21.3	0.018	0.082	0.351
** d 25 to 28**	182	181	188	20.3	0.970	0.691	0.669
** d 21 to 28**	146	164	161	10.5	0.153	0.223	0.826
** d 29 to 49**	362	381	373	16.4	0.075	0.296	0.402
** d 21 to 49 (overall)**	307	326	319	13.5	0.049	0.198	0.466
**ADFI (d 21 to 24), g**	103	133	116	17.1	<0.001	0.020	0.002
**G:F (d 21 to 24)**	0.92	1.03	1.07	0.09	0.383	0.234	0.753
**Pen CV, %^b^**							
** d 21 (weaning)**	23.8	25.1	23.5	0.82	0.263	0.787	0.167
** d 24**	22.3	22.5	21.3	0.91	0.835	0.425	0.294
** d 28**	22.9	21.8	21.2	0.95	0.408	0.193	0.632
** d 49**	20.7	19.5	19.6	0.99	0.409	0.431	0.969
**CV change, %^c^**							
** d 21 to 24**	−1.6	−2.5	−2.3	0.86	0.391	0.522	0.824
** d 21 to 28**	−0.9	−3.2	−2.3	0.83	0.062	0.263	0.437
** d 21 to 49**	−3.0	−5.6	−3.1	0.89	0.031	0.961	0.022

a A total of 675 pigs (initially 6.4 kg and 21.2 ± 0.2 d of age, C29 × 327 PIC), with 25 pigs per pen and 9 replications per treatment. Group composition: non-eater (N) = no-creep fed pigs and non-eaters, creep (C) = eaters, and mixed (M) = 52% non-eaters and 48% eaters.

b Pen CV = coefficient of variation within pen.

c CV change = difference in pen CV between two time points; final %CV—initial %CV.

These results are consistent with previous findings (Bruininx et al 2002; Kim et al 2005; Kuller et al 2007; Pluske et al 2007; Sulabo et al 2010b, 2010c). Bruininx et al (2002) provided the first evidence that eaters had greater daily gains than non-eaters and pigs not provided creep feed. Likewise, Kim et al (2005) and Pluske et al (2007) observed differences in initial post-weaning daily gains between creep feed consumption categories, where “good” eaters grew faster immediately after weaning than piglets classified as moderate or poor eaters. The difference in post-weaning feed intake between creep feed eaters and non-eaters also has been consistent (Fraser et al 1994; Delumeau and Meunier-Salaün 1995; Bruininx et al 2002, 2004; Carstensen et al 2005; Sulabo et al 2010d), although one study revealed different findings (Hedemann et al 2007). Pigs in most previous studies were provided creep feed for 14 to 21 d and weaned at an older age (ranging from 24 to 31 d). Pigs in the current study had a shorter creep feeding duration (3 d prior to weaning) and were weaned at a younger age (21 d). These results suggest that individual pigs that consume creep feed prior to weaning consume more feed and achieve higher daily gains post-weaning even when creep fed for a short duration and weaned at 3 wk of age. However, it is not known if the same responses can be expected for pigs weaned earlier (<3 wk).

**Fig. 3.**
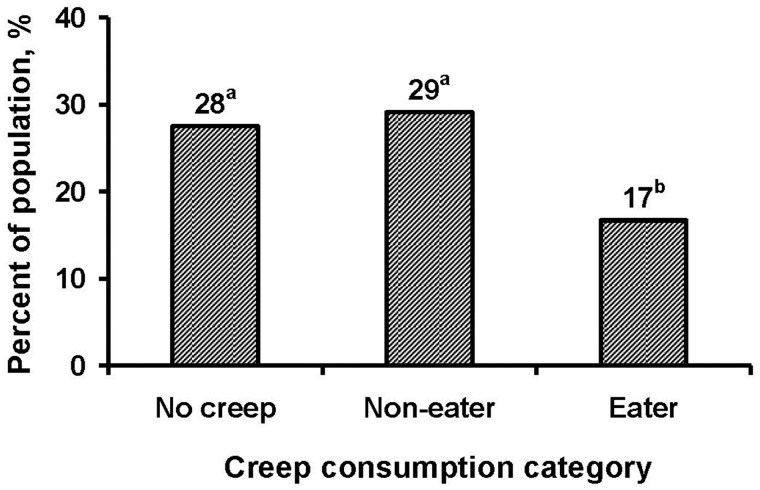
Percentage of pigs that did not gain weight or lost weight in the first 3 d postweaning. No creep = pigs that were not provided creep feed pre-weaning, non-eater = pigs offered creep feed but did not consume creep feed, eater = pigs that consumed creep feed. Category effect,^a,b^*P* < 0.001.

At d 21 (weaning), there were no differences in initial pen CV between the 3 groups (Table 6). There were no differences in pen CV at d 24, 28, and 49; however, the reduction in pen CV in the eater group tended to be greater (−3.2% vs. −0.9%; *P *= 0.062) at d 28 than in the non-eater group, with the mixed group intermediate. Overall (d 21 to 49), the change in pen CV for the eater group was greater (−5.6%; *P *= 0.031) than in both the non-eater and mixed groups. Studies that evaluated the effect of creep feed on within pen CV are scarce, but like the present study, Middelkoop et al (2020) observed a reduction in BW variation of 1.5% with creep feeding. These results suggest that individual consumption characteristics of pigs prior to weaning may be an important factor in improving pig weight uniformity in the nursery. The greater reduction in weight variation in the eater groups may be driven by faster growth of smaller pigs, especially during the first week post-weaning. In addition, the percentage of pigs that lost weight in the first 3 d post-weaning was reduced (*P *< 0.001) for eaters at 17%, while 28% of no-creep fed pigs and 29% of non-eaters lost weight (Fig. 3). This indicates that consumption of creep feed pre-weaning can reduce the post-weaning lag, despite a large proportion of eaters being smaller at weaning than non-eaters and no-creep fed pigs.

Social facilitation is a rudimentary form of social learning in which individuals discover resources by following group members that have already learned to exploit these resources (­Giraldeau and Caraco 2000). Nicol and Pope (1994) and Held et al (2000) both demonstrated that inexperienced pigs could be directed to the location of food by an experienced pig. If social facilitation occurs, transmission of information regarding locating and consuming a new food source between experienced (eaters) and inexperienced (non-eaters) pen mates may be important in reducing pigs with low feed intake and improving weaning transition. Overall nursery performance of the mixed group was mostly intermediate to that of the eater and non-eater groups. However, the best criterion is to evaluate performance of individual non-eaters and eaters within the mixed pens. In the mixed pens at d 21 (weaning), eaters had 0.45 kg less (*P *= 0.020) BW than non-eaters (Table 7). From d 21 to 24, eaters had greater (*P *< 0.001) ADG than non-eaters. This resulted in a 62% reduction (0.45 to 0.17 kg) in the BW difference between eaters and non-eaters after 3 d post-weaning. From d 25 to 28, there were no differences; however, eaters had greater (*P *= 0.002) ADG from d 21 to 28 and d 29 to 49 (*P *= 0.045) and overall (d 21 to 49, *P *= 0.007) than non-eaters. In fact, the performance of eaters and non-eaters in the mixed pens was similar to the performance of eaters and non-eaters penned separately. This suggests that social facilitation did not occur between eaters and non-eaters. These results were not consistent with the findings of Morgan et al (2001) but concur with those of Reynolds et al (2007). Using pairs of pigs, Morgan et al (2001) observed that the presence of an experienced piglet stimulated feed intake of a pair of piglets housed in the same pen and stimulated the initial feeding behavior of the inexperienced piglet. In another study, Reynolds et al (2007) observed that the latency time of inexperienced piglets was longer when these piglets were mixed with experienced (creep fed) piglets, which did not support the hypothesis of social facilitation between piglets differing in exposure to creep feed.

**Table 7.** Post-weaning growth performance of non-eater and eater pigs within mixed pens.^a^

Item	Consumption		SEM	*P*-value
	Non-eater	Eater		
**N**	117	108	…	…
**BW, kg**				
**d 21**	6.72	6.27	0.14	0.019
** d 24**	6.92	6.75	0.14	0.388
** d 28**	7.73	7.52	0.15	0.355
** d 49**	15.16	15.43	0.37	0.542
**ADG, g**				
** d 21 to 24**	69	162	16.1	<0.001
** d 25 to 28**	202	192	11.4	0.489
** d 21 to 28**	145	179	10.1	0.002
** d 29 to 49**	354	377	13.7	0.045
** d 21 to 49 (overall)**	302	328	11.4	0.007

a A total of 675 pigs (initially 6.4 kg and 21.2 ± 0.2 d of age, C29 × 327 PIC), with 25 pigs per pen and 9 replications per treatment were used. Group composition: 52% non-eaters (no-creep fed pigs and creep fed non-eaters, 13 pigs per pen) and 48% eaters (12 pigs per pen). In the mixed treatment, differences between non-eaters and eaters were analyzed with pen as the block and pig as the experimental unit.

When evaluating the subpopulations of eaters and non-eaters for within treatment for the overall study (Table 8), pigs identified as eaters were lighter (*P *< 0.001) at both d 18 and 21 (weaning) than creep fed non-eaters regardless of the creep feed quality. The BW of pigs not provided creep feed were intermediate. Due to the short period and slight differences in BW, there was no evidence for difference in pre-weaning ADG from d 18 to 21. From d 21 to 24, eaters of the complex and simple creep feed had greater (*P *< 0.05) ADG than non-eaters of creep feed. However, only complex feed eaters had greater (*P *< 0.05) gain than no creep pigs, even though simple creep feed eaters had numerically greater ADG than no creep pigs (131 g vs. 95 g). In the following periods and for the overall nursery stage of the study, no differences in ADG were observed between eaters and non-eaters from both creep feed qualities and no creep feed pens.

**Table 8.** Pig performance according to creep consumption category from lactation to 28 d post-weaning.^a^^,^^b^

Item	No creep	Simple creep feed		Complex creep feed		SEM	*P*-value
		Non-eater	Eater	Non-eater	Eater		
**N**	285	203	79	145	304	…	…
**Pig BW, kg**							
** d 0 (post-fostering)**	1.53	1.50	1.51	1.52	1.50	0.08	0.895
** d 18 (start creep)**	5.68^cde^	5.74^cd^	5.39^e^	5.92^c^	5.53^de^	0.21	<0.001
** d 21 (weaning)**	6.43^cde^	6.47^cd^	6.09^e^	6.74^c^	6.29^de^	0.23	<0.001
** d 24**	6.66	6.64	6.48	6.91	6.67	0.24	0.273
** d 28**	7.40	7.36	7.18	7.64	7.39	0.26	0.277
** d 49**	14.74	14.87	14.93	15.29	15.03	0.51	0.831
**ADG, g**							
** d 18 to 21**	288	279	273	318	301	0.14	0.112
** d 21 to 24**	95^cd^	80^c^	131^de^	75^c^	135^e^	0.20	<0.001
** d 24 to 28**	175	160	176	170	180	0.14	0.509
** d 28 to 49**	352	360	369	361	367	0.13	0.703
** d 21 to 49 (overall)**	299	301	316	305	315	0.11	0.245

a n = 1016 pigs (C29 × 327 PIC) were used for the analysis.

b Pig categories: no creep = pig not provided creep feed during lactation, simple = pig provided a simple creep diet during lactation, complex = pig provided a complex creep diet during lactation, non-eater = pig tested negative for green-colored feces, eater = pig tested positive for green-colored feces. Data were analyzed with pig as the experimental unit.

c,d,e Row values (least square means ± SE) with different superscripts differ (*P *< 0.05).

Studies evaluating the creep feed quality on nursery performance have generated interesting but contradictory findings. Like the current study, Sands et al (2022) observed an improvement in growth performance in the first days post-weaning, even though the difference was not maintained throughout the nursery period regardless of the creep feed quality. Research investigating the interaction between creep and nursery diet quality concluded that the pre-weaning diet has a limited influence on post-weaning performance (Christensen and Huber 2021). On the other hand, Collins et al (2013) observed greater nursery ADG for pigs fed a simpler creep feed for pigs weaned at 22 and 29 d of age. The simple creep feed in the Collins et al (2013) study had a significant amount of complex ingredients, such as bloodmeal, acidifiers, tallow, oats, and zinc oxide, different from simple vs. complex creep feed diets in most research. Previous familiarity with a diet can also affect early nursery performance. Pigs fed the same diet pre- and post-weaning had improved ADG in the first 14 d in the nursery compared to those fed different diets, regardless of diet complexity (Heo et al 2018). These results indicate that incorporating costly, highly digestible ingredients may not consistently enhance post-weaning performance compared to a simple creep feed. The outcomes of creep feed quality will depend on factors such as wean age and nursery feed regimen, and possibly other variables not yet identified.

In summary, creep feed quality affects pre-weaning BW gains, litter creep feed disappearance, and the proportion of eaters. Eaters had lower pre-weaning gains and lighter weaning weights and tended to nurse more in the middle and posterior teats compared with non-eaters. Individual creep feed consumption characteristics influenced post-weaning feed intake, daily gains, weight uniformity, and post-weaning lag. Social facilitation did not occur in weaned pigs housed in large commercial groups. The benefit of pre-weaning creep consumption on post-weaning performance was independent of diet complexity.

## Acknowledgement

Contribution no. 25-099-J from the Kansas Agricultural Experiment Station, Manhattan, KS 66506-0201, United States.
